# The chemopreventive retinoid 4HPR impairs prostate cancer cell migration and invasion by interfering with FAK/AKT/GSK3β pathway and β-catenin stability

**DOI:** 10.1186/1476-4598-9-142

**Published:** 2010-06-10

**Authors:** Roberto Benelli, Stefano Monteghirfo, Roberta Venè, Francesca Tosetti, Nicoletta Ferrari

**Affiliations:** 1 Oncologia Molecolare e Angiogenesi, Istituto Nazionale per la Ricerca sul Cancro, Largo R.Benzi 10, 16132 Genova, Italy; 2 Biologia Cellulare, Istituto Nazionale per la Ricerca sul Cancro, Largo R.Benzi 10, 16132 Genova, Italy

## Abstract

**Background:**

Prostate cancer shows an extremely slow progression, appearing in its metastatic, hormone refractory phenotype mostly in elderly men. The chemopreventive targeting of this tumor could accordingly delay its malignancy over life expectancy. The cancer chemopreventive retinoid *N*-(4 hydroxyphenyl)retinamide (4HPR) has already been shown to restrain prostate cancer growth in vitro and in vivo, though its mechanisms of action are only partially explained.

**Results:**

We found that 4HPR impairs DU145 and PC3 prostate cancer cells migration and invasion by down-regulating FAK and AKT activation and by enhancing β-catenin degradation, causing the downregulation of target genes like cyclin D1, survivin and VEGF. This non-migratory phenotype was similarly produced in both cell lines by stable silencing of β-catenin. 4HPR was able to decrease AKT phosphorylation also when powerfully upregulated by IGF-1 and, consequently, to impair IGF-1-stimulated cell motility. Conversely, the expression of constitutively active AKT (myr-AKT) overcame the effects of 4HPR and β-catenin-silencing on cell migration. In addition, we found that BMP-2, a 4HPR target with antiangiogenic activity, decreased prostate cancer cell proliferation, migration and invasion by down-regulating the pathway described involving AKT phosphorylation, β-catenin stability and cyclin D1 expression.

**Conclusion:**

These data point to 4HPR as a negative regulator of AKT phosphorylation, effectively targeting the β-catenin pathway and inducing a relatively benign phenotype in prostate cancer cells, limiting neoangiogenesis and cell invasion.

## Background

Prostate cancer (PC) is the most frequent cancer in men of western countries. About 1 man in 5 is diagnosed with PC during his lifetime and 1 man in 33 will die of this disease. As the population age is increasing, these numbers are expected to increase. PC cells usually remain confined in the organ, while a small proportion of carcinomas acquire the ability to metastasize and approximately 80% of patients who have died of advanced hormone refractory PC have clinical evidence of bone metastasis. Early stage disease differs from later stages in tumor volume, localization and metastatic potential. Processes involved in later stage disease, like development of androgen independence as a consequence of androgen depletion therapy, neoangiogenesis and homing of metastatic cells in lymphatic or bone tissues are generally undetectable at early stages. Among control strategies, chemoprevention attempts in preclinical studies to halt or delay these processes are now proving the potential efficacy of this approach.

4HPR, also known as fenretinide, has received great attention as a chemopreventive agent based on the cumulative results of numerous in vitro and animal studies, as well as chemoprevention clinical trials [1]. 4HPR administration prevents prostate tumor growth and metastasis in animals [2-6] and functions as an apoptosis inducer in human prostate cancer cells in vitro [7-9] mostly through the production of reactive oxygen species (ROS) and mitochondrial disruption [1]. Interestingly, 4HPR was shown to lower circulating insulin-like growth factor I (IGF-I) levels which have been associated with a higher risk of prostate cancer in several cohort studies [10,11].

We and others have previously reported that the chemopreventive effects of 4HPR in early intervention protocols are likely due to its antiangiogenic properties [4,12-16]. Since angiogenesis and metastatic spread are strictly related, in this study we analyzed the regulation of multiple signaling pathways responsible for cancer cell invasion. We found that 4HPR-induced inhibition of PC cell migration and invasion correlates with decreased FAK and AKT phosphorylation, activation of the glycogen synthase kinase 3β(GSK3β) and β-catenin destabilization. As a consequence, 4HPR led to the regulation of genes controlling cell proliferation, angiogenesis and metastasis. For the slow evolving prostate tumor to become metastatic multiple mechanisms must be activated and our results identify novel points of regulation by 4HPR, independent of ROS generation, that further support its use as a chemopreventive or therapeutic agent. Due to its pleiotropic activities, 4HPR could be used alone, with other chemopreventive molecules exhibiting complementary mechanisms of action, or in combination with chemotherapy to treat prostate cancer.

## Methods

### Cell culture and reagents

Androgen-independent DU145 and PC3 prostate carcinoma cell lines (ATCC, Rockville, MD) were cultured in RPMI containing 10% heat-inactivated FCS. DU145 subclones resistant to 5 μM 4HPR (R5) were established from cultures progressively exposed to increasing concentrations of 4HPR starting from 1 μM and cloning by limiting dilution following published procedures [17]. 4HPR (kindly provided by Dr. James A. Crowell, Division of Cancer Prevention, NCI, Bethesda and Dr. Gregg Bullard, McKessonBio, Rockville, MD) was dissolved in ethanol at a stock concentration of 10 mM and stored in aliquots at -20°. Wortmannin, LY294002, *N*-Acetyl-L-cysteine (NAC) and diphenyleneiodionium chloride (DPI) were from Sigma (Milano, Italy), IGF-1 and bone morphogenetic protein-2 (BMP-2) were from R&D (Minneapolis, MN, USA). VEGF protein released into the media by PC cells was measured using a commercial human ELISA kit (Biosource, Invitrogen, Carlsbad, CA, USA).

### Cell proliferation, apoptosis, chemotaxis and invasion assays

*In vitro *cell proliferation was performed on cells plated in 96-well plates at 3,000 cells/well dilution and grown in complete medium or treated as described. The medium was changed every two days. At different time points, the number of viable cells was evaluated by the crystal violet assay.

Chemotaxis and chemoinvasion assays were carried out in Boyden chambers as previously described [18]. The cells (12 × 10^4^/chamber) were extensively washed with PBS, resuspended in serum-free media (SFM) and placed in the upper compartment with or without selected molecules. In parallel experiments, trypan blue exclusion under all the conditions tested showed no altered cell proliferation or viability compared with controls during the five hours of chemotaxis test. The two compartments of the Boyden chamber were separated by 8 μm pore-size polycarbonate filters coated with 5 μg/50 μl/filter of collagen type IV (diluted in H_2_O, 0.1% CH_3_COOH) for the chemotaxis assay, or with Matrigel (60 μg/filter), a reconstituted basement membrane, for the invasion assay. Serum free 24 h-conditioned medium from human fibroblasts (FB-CM) was used as a chemoattractant in the lower chamber. After 5 hours of incubation at 37° in 5% CO_2_, the filters were recovered, fixed in ETOH and stained by Toluidine blue after removal of the non-migrating cells on the upper surface. The migrated cells were quantified counting five to ten fields for each filter under a microscope. Graphical results are shown as percent inhibition as compared to a 100% untreated control.

### Protein extraction and western blot analyses

Proteins were obtained from PC cells after four hours culture in the absence or presence of the indicated molecules as described in the text and in figure legends, or after 4 days in the presence of BMP-2 (50-100 ng/ml). To perform AKT activation, thirty min before the end of the incubation, the cells were stimulated with IGF-I (100 ng/ml). The cells were then lysed in RIPA buffer containing protease inhibitors. Protein concentration was determined with the DC Protein Assay kit (Bio-Rad). Equal amounts of samples were resolved by SDS-PAGE, transferred to nitrocellulose and probed at 4°C overnight with the following anti-human antibodies (Cell Signaling Technology, Beverly, MA): rabbit polyclonal anti-phospho-FAK (Tyr576/577), phospho-AKT-1 (Ser473), phospho-GSK3β (Ser9), β-catenin, phospho-β-catenin (Ser552), cyclin D1, survivin and E-cadherin. After washing, the blots were incubated for 1 h at room temperature with horseradish peroxidase-conjugated secondary antibodies (GE-Healthcare, Milano, Italy) and specific complexes were revealed by enhanced chemiluminescence (ECL, GE-Healthcare). An anti-GAPDH antibody conjugated to horseradish peroxidase (Novus Biologicals, Littleton, CO) or a mouse monoclonal anti-β-tubulin antibody (Sigma, Milano, Italy) were utilized as loading controls for all samples.

### Plasmid DNA, RNA silencing and transfections

pCMV6-Myr.Akt (a constitutively active Akt) and control vector were kindly provided by Dr. Alex Toker of the Beth Israel Deaconess Medical Center of Boston. Transient transfections were performed with lipofectamine 2000 (Invitrogen). β-catenin was silenced using a lentiviral vector expressing short hairpin RNA (MISSION shRNA clones, Sigma) following manufacturer's instructions. This resulted in the generation of stable DU145 and PC3 cells showing decreased β-catenin expression. Control vectors contained scrambled sequences.

### Statistical analysis

Data are expressed as means ± SD. The statistical significance between two data sets was determined by two-tailed unpaired Student's *t *test using the PRISM GraphPad software.

## Results

### 4HPR decreases cell proliferation, migration and invasion

**Figure 1:**
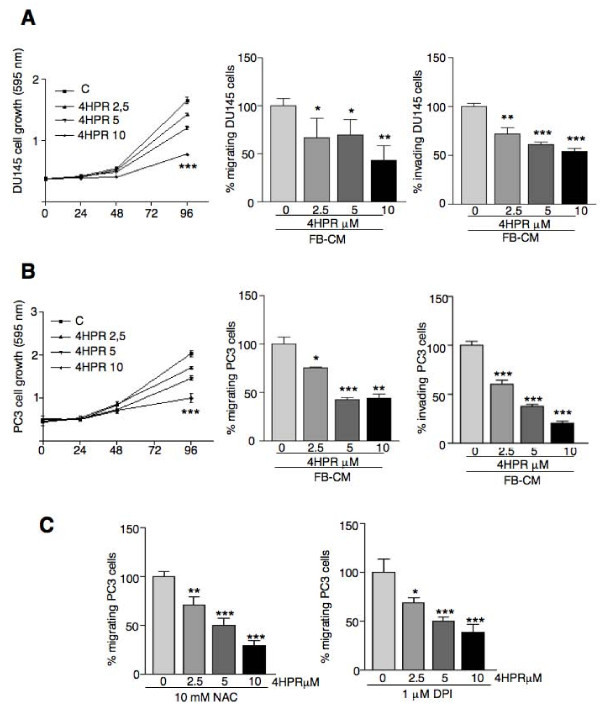
**Inhibition of androgen-independent DU145 (A) and PC3 (B) prostate cancer cell growth, migration and invasion by increasing doses of 4HPR is independent of ROS production (C)**. Cell proliferation, evaluated by the crystal violet assay, is significantly inhibited at all 4HPR concentrations tested after 96 h exposure (***P < 0.001). In migration and invasion assays, the conditioned medium from human fibroblasts (FB-CM) was used as chemoattractant in the lower chamber. Experiments were done in triplicate and repeated thrice. Means ± SD are shown (*P < 0.05, **P < 0.01, ***P < 0.001).

We first assessed the action of 4HPR on prostate cancer cell proliferation by treating DU145 and PC3 cells with a range of concentrations. All the concentrations tested inhibited cell growth, with statistically significant differences only after 96 h exposure (Fig 1A, B left panels).

Migration of cancer cells is one of the key factors responsible for cancer metastasis. To metastasize, cancer cells must migrate from the original growth site, invade surrounding tissues and locate to other parts of the body through the blood or the lymphatic system. The effects of 4HPR on PC cell migration and invasion were then tested in a broad dose/response experiment. Untreated PC cells migrated (Fig. 1A, B middle; DU145 and PC3 respectively) and invaded through Matrigel (Fig. 1A, B right; DU145 and PC3 respectively) in response to fibroblast conditioned medium (FB-CM). A very short exposure (5 h) to micromolar concentrations of 4HPR significantly inhibited migration and invasion of DU145 (Fig. 1A) and PC3 (Fig. 1B) cells. To explore the possibility that ROS induction by 4HPR was a mechanism underlying these effects, PC3 cells were pretreated 1 h with the ROS scavengers NAC and DPI (at 10 mM and 1 μM respectively) and then subjected to chemotaxis in the presence of 4HPR at different concentrations. As shown in Fig. 1C, both compounds did not modify 4HPR effects. Additional Fig. 1 (Fig. 1S) shows that 30 min pretreatment with the ROS scavenger N-Acetyl Cysteine (NAC) at 10 mM significantly decreases ROS production induced by 4HPR treatment (1 h at 5 μM).

### 4HPR modulates biological responses involved in the metastatic process of prostate cancer

**Figure 2:**
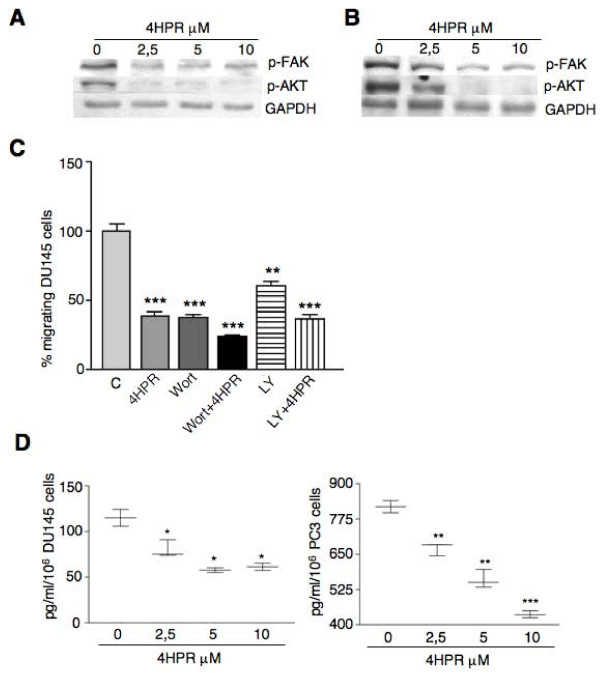
**4HPR inhibits FAK and AKT activity in prostate cancer cells, thus controlling their migratory potential and VEGF release**. Western blot analyses show a remarkable decrease of FAK and AKT phosphorylation after 4 h of treatment with 4HPR in DU145 (A) and PC3 (B) cells. C). The specific PI3K/AKT inhibitors Wortmannin (Wort, 200 nM) and LY294002 (LY, 10 μM) significantly impair the chemotaxis of DU145 cells towards FB-CM similar to 4HPR (5 μM). Co-treatment with Wortmannin and 4HPR produces an even more pronounced effect. Similar results were obtained with PC3 cells. Experiments were done in triplicate and repeated thrice. Comparisons were made with control cells. Means ± SD are shown (***P < 0.001). D). Decreased AKT activity induced by 4HPR correlates with reduced VEGF secretion by DU145 and PC3 exposed for 16 h to the drug and analyzed by ELISA. Means ± SD are shown (*P < 0.05, **P < 0.01, ***P < 0.001).

Focal adhesion kinase (FAK) is a non-receptor tyrosine kinase that plays an important role in signal transduction and is a key regulator of survival, proliferation, migration and invasion. Overexpression and/or increased activity of FAK are common in a wide variety of human cancers, implicating a role for FAK in carcinogenesis. DU145 and PC3 cells express high levels of activated FAK, which was rapidly (4 hours) downregulated by 4HPR (Fig. 2A, B). The survival and migratory signaling mediated by FAK operates via activation of the PI3K/AKT pathway [19,20] that in turn promotes prostate cancer cell migration and invasion [21]. Exposure to 4HPR rapidly decreased AKT phosphorylation in both cell lines (Fig. 2A, B). In agreement, prostate cancer cell migration towards FB-CM was significantly inhibited in the presence of the specific PI3K inhibitors Wortmannin (Wort, 200 nM) and LY294002 (LY, 10 μM). Co-exposure of the inhibitors with 4HPR produced more pronounced effects being the combination Wotmannin/4HPR the most effective. (Fig. 2C, DU145 cells; PC3 cells gave similar results). These data suggest that these pathways are partially independent of each other. As AKT overexpression correlates with increased VEGF levels and prostate tumor angiogenesis [22], we determined VEGF release in 4HPR-treated cells by ELISA. We noted that 4HPR-induced AKT downregulation is associated with reduced VEGF secretion (Fig. 2D).

**Figure 3:**
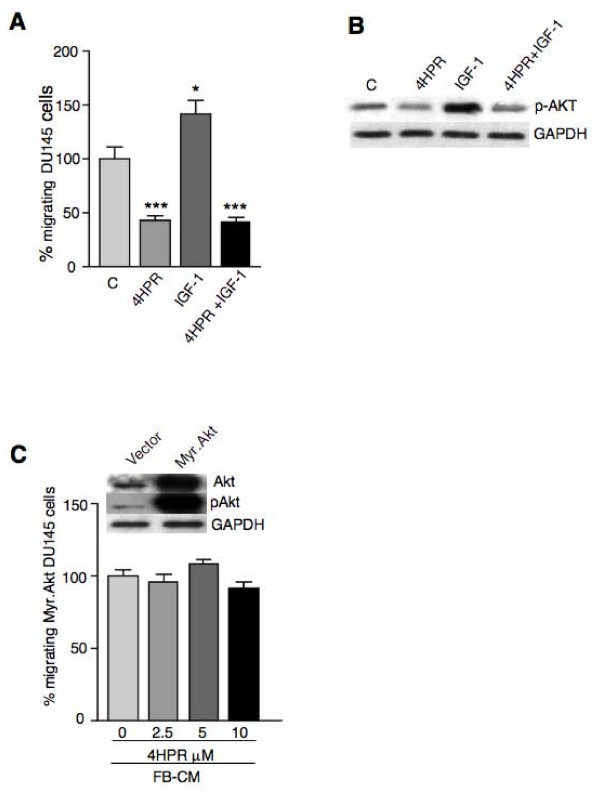
**4HPR antagonizes AKT activation by IGF-I but not constitutively active AKT**. A). DU145 cell migration towards FB-CM is significantly enhanced by IGF-I (100 ng/ml), as compared to control cells. IGF-I effects are completely abrogated when cell migration is carried out in the presence of 4HPR (5 μM). Experiments were done in triplicate and repeated thrice. Means ± SD are shown (*P < 0.05, ***P < 0.001). B). Western blot analysis of extracts from DU145 cells pulsed for 30 min with IGF-I at 100 ng/ml show strong AKT phosphorylation, almost completely abolished by 4 h pretreatment with 5 μM 4HPR. C). DU145 cells transiently transfected with a constitutively active form of AKT (Myr.Akt) and showing high levels of pAKT as compared to vector transfected control cells (inset) are no more susceptible to 4HPR-induced inhibition of cell migration. PC3 transfected cells produced similar results.

The AKT activator IGF-1 is a potent mitogenic and motogenic factor and has a prominent role in protection against apoptosis and cell survival. IGF-1 has been implicated in the initiation and progression of several different cancers including prostate cancer [23]. Chemotaxis assays showed that IGF-1 stimulates androgen-independent DU145 prostate cancer cell migration and co-exposure to 4HPR completely abrogated the effect (Fig. 3A). Accordingly, activation of the AKT signaling pathway by a short exposure to IGF-I (30 min at 100 ng/ml), as detected by western blot analysis, was lowered by 4HPR pretreatment (Fig. 3B).

Next, we determined the effect of overexpression of myristoylated Akt (Myr.Akt), which is anchored to the plasma membrane and has a constitutively active kinase activity. Cells transfected with the empty vector were used as controls. Western blot analysis of extracts from DU145 cells transiently transfected with constitutively active Akt showed high levels of total Akt and phosphorylated (Ser 473)-Akt as compared with the empty vector-transfected control cells (Fig. 3C). Ectopic expression of constitutively active Akt abrogated 4HPR-mediated inhibition of DU145 cell migration (Fig. 3C).

### The oncogenic hub β-catenin is a molecular target of 4HPR

**Figure 4:**
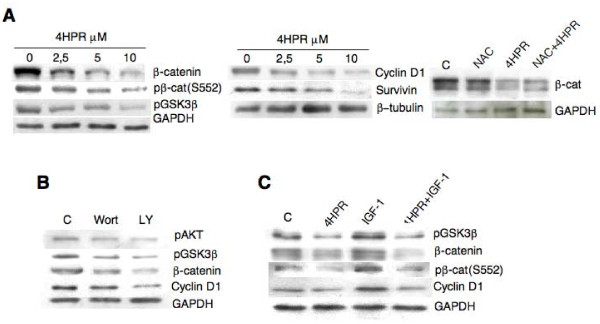
**4HPR regulates the β-catenin soluble pool, its transcriptionally active phosphorylated (Ser522) form, GSK3β phosphorylation, cyclin D1 and survivin expression**. A). Dose-dependent decrease of β-catenin and phospho-β-catenin (Ser522), and GSK3β- phosphorylation after 4 h of treatment in DU145 cells (left) is associated with reduced levels of the proliferation/survival related genes cyclin D1 and survivin (middle). Loss of β-catenin stability is independent of ROS production as demonstrated by incubating the cells in the presence of the ROS scavenger NAC (right). B). Similar to 4HPR, 4 hours incubation with the specific PI3K/AKT inhibitors wortmannin (Wort, 200 nM) and LY294002 (LY, 10 μM) activate GSK3β and reduce the soluble pool of β-catenin and cyclin D1 levels. C). IGF-I (4 hours at 100 ng/ml), through AKT activation, causes inhibition of its down-stream effector GSK3β, thus leading to β-catenin stabilization, β-catenin (ser522) phosphorylation and cyclin D1 accumulation. 4HPR (5 μM) co-exposure can still antagonize IGF I-induced AKT activity and reduce β-catenin and cyclin D1 levels. The experiments were repeated thrice and similar results were obtained with PC3 cells.

β-catenin is a multifunctional protein not only involved structurally in the adherens junction complex, but also acting as a signaling molecule promoting cancer cell proliferation, survival and migration. β-catenin signaling is aberrantly activated in greater than 70% of colorectal cancers and has been shown to play a causative role in prostate cancer [24]. As β-catenin turnover is triggered by GSK-3β, a well known target of AKT, we made β-catenin signaling a focal point of our investigation. We measured the soluble levels of β-catenin by western blot analysis in 4HPR-treated DU145 cells (Fig. 4A, left) and evaluated cyclin D1 and survivin as typical transcriptional targets of the β-catenin/TCF/LEF complex [25,26] (Fig. 4A, middle). As shown in Figure 4A, DU145 cells exhibited high levels of β-catenin, cyclin D1 and survivin expression. A short exposure to 4HPR (4 hours) resulted in a significant reduction in the levels of these proteins. Pre-incubation of cells with the ROS scavenger NAC (1 h at 10 mM) did not compromise the ability of 4HPR to modulate β-catenin thus suggesting a redox-independent signaling (Fig. 4A right). Similar results were obtained with PC3 cells (data not shown). These data suggest that the highly activated β-catenin signaling was suppressed by short-term 4HPR treatment in prostate tumor cells.

The expression of β-catenin is rather low in non-cancer cells as GSK-3β causes its phosphorylation and consequent degradation by the ubiquitin-proteasome pathway. GSK-3β activity is suppressed when it is phosphorylated on serine 9 by AKT. In agreement with the decreased phosphorylation of AKT after 4HPR treatment (Fig. 2A, B, 3B), GSK-3β phosphorylation was decreased by 4HPR (Fig. 4A, left). As a confirmation, a short exposure (2 hrs) to the specific PI3K/AKT inhibitors Wortmannin and LY294002 reduced GSK3β phosphorylation,β-catenin and cyclin D1 levels (Fig. 4B). AKT was also shown to directly phosphorylate β-catenin at Ser552, independent of GSK3β. Phosphorylation at Ser552 induces β-catenin accumulation in the nucleus, increases its transcriptional activity and promotes cell invasion [27,28]. According to AKT inhibition by 4HPR exposure, β-catenin phosphorylated at Ser552, probably representing the residual nuclear pool escaping proteasomal degradation, was decreased (Fig. 4A, left). Activation of AKT by IGF-I exposure (Fig. 4C) suppressed GSK3β activity, stabilized β-catenin, increased Ser552 phosphorylation and cyclin D1 levels, yet pretreatment with 4HPR abolished the effects of IGF-I stimulation (Fig. 4C). Together, all these data suggest that 4HPR-induced pAKT down-regulation exerts a multi-level control on total and nuclear β-catenin, dependent and independent of GSK3β activity.

In order to understand whether the observed signaling was univocally related to sensitivity of cell lines to 4HPR, our analysis was extended to DU145/R5, a cell line resistant to 5 μM 4HPR generated by in vitro incubation of DU145 cells with increasing concentrations of 4HPR. Supplementary Fig. 2 (Fig. 2S) shows that DU145/R5 cells grow (96 h treatment, panel A) and migrate (5 h treatment, panel B) in the presence of 2.5 and 5 μM 4HPR. This 4HPR-resistant phenotype is associated to very high levels of phosphorylated AKT (panel C).

### Effect of β-catenin silencing in human prostate cancer DU145 and PC3 cells

**Figure 5:**
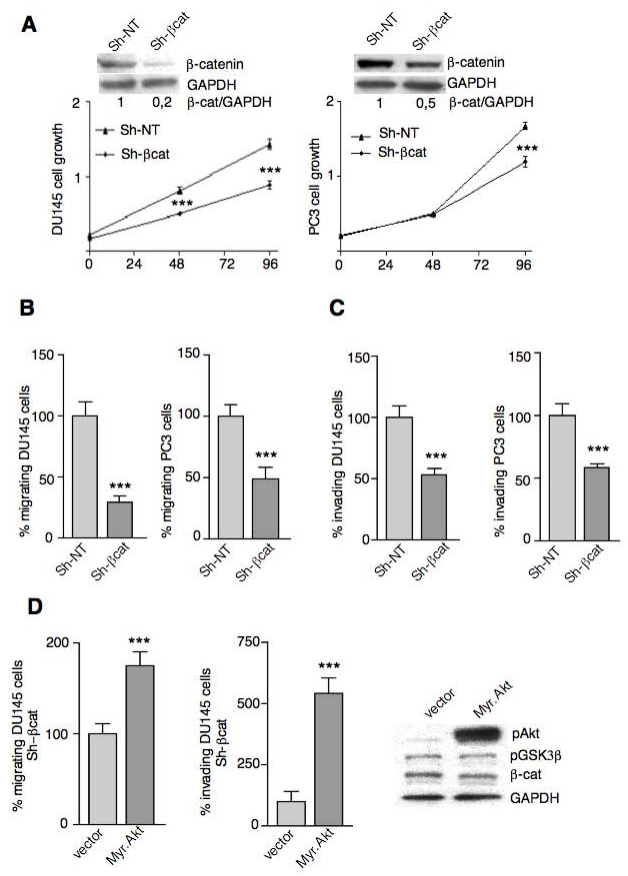
**β-catenin levels control prostate cancer cell proliferation, migration and invasiveness**. A). DU145 and PC3 cells permanently transfected with β-catenin shRNA sequences (Sh- βcat) had decreased levels of β-catenin as compared to non-target shRNA control vector (sh-NT) transfected cells (insets) and showed decreased cell proliferation as evaluated by the crystal violet assay. β-catenin silenced DU145 and PC3 cells had impaired ability to migrate toward FB-conditioned medium (B) and to invade through matrigel (C). D). Sh- βcat DU145 cells were transiently cotransfected with activated AKT (Myr.Akt) or empty vector (vector) and 24 hours after transfection assayed for their ability to migrate toward FB-conditioned medium or to invade through matrigel. Although AKT activation greatly nullify the effects of β-catenin silencing on cell migration and invasion, activation of Akt alone was not able to phosphorylate and inhibit the GSK3β pool involved in β-catenin up-regulation, thus β-catenin levels remained unaltered (right panel). All results are representative of three independent experiments (***P < 0.001).

We first investigated whether specifically reducing the levels of β-catenin resulted in decreased proliferation, migration and invasiveness. For this analysis, RNA interference with shRNAs directed against β-catenin (sh-βcat) was used and comparisons were made with controls with scrambled sequences (sh-NT). As shown in Fig. 5, shRNA targeting of β-catenin (insets panel A) resulted in a 40% and 30% decrease in cell proliferation seen at 96 h (Fig. 5A, DU145 and PC3 cells respectively). β-catenin silencing significantly reduced also cell migration (Fig. 5B, DU145 and PC3 cells respectively) and invasiveness (Fig. 5C, DU145 and PC3 cells respectively).

Next, we determined whether constitutively active Akt (Myr.Akt) could abrogate the effects of β-catenin silencing on cell migration and invasion. β-catenin-silenced DU145 cells transiently expressing Myr.Akt showed an enhanced migratory and invasive phenotype as compared to control vector transfected cells (Fig. 5D). In these cells GSK3β phosphorylation and β-catenin levels remained unaltered (Fig. 5D, right panel), as previously reported [29], suggesting that under these conditions activation of Akt alone is not able to restore β-catenin levels through increased expression or stabilization.

### 4HPR-induced BMP2 in an anti-angiogenic setting antagonizes prostate cancer cell growth and invasiveness

**Figure 6:**
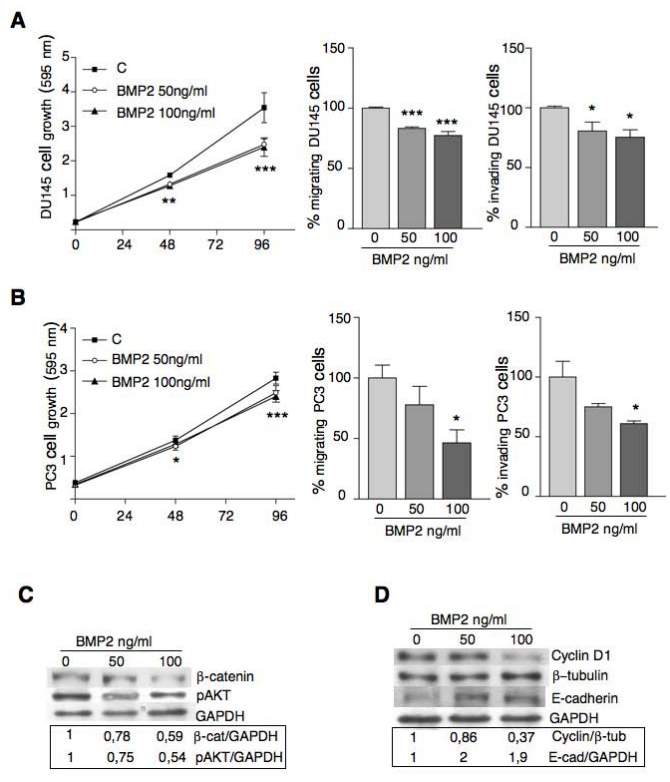
**Effects of 4 days administration of BMP-2 (50-100 ng/ml) on growth, migration, invasion and transcriptional activity of DU145 and PC3 cells**. Cell growth, as evaluated by the crystal violet assay, is significantly inhibited by BMP-2 at 48 and 96 h (A, B, left panels, DU145 and PC3 cells respectively). Means ± SD of three independent experiments run in sextuplicate are shown (*P < 0.05, **P < 0.01, ***P < 0.001). DU145 and PC3 cells exposed for 4 days to 50-100 ng/ml BMP-2 and then subjected to the chemotaxis assay show a decreased migratory activity (A, B, middle panels) that was associated with a less invasive/metastatic phenotype (A, B, right panels). Means ± SD of three independent experiments run in triplicate are shown (*P < 0.05, ***P < 0.001). C). DU145 cells exposed for 4 days to BMP-2 (50-100 ng/ml) show significant reduction of soluble β-catenin that correlates with less active AKT, reduced cyclin D1 levels (D) and increased expression of E-cadherin (D), all indicative of a less metastatic phenotype. The experiments were carried out independently two times and PC3 gave similar results. Protein expression, relative to controls set at 1, is shown.

Metastasis and angiogenesis are strictly related processes. We reported that the TGF-β family member BMP-2 is a mediator of the antiangiogenic activity of 4HPR [14], controlling tumor growth. Since the role of BMPs in the formation of prostate cancer metastases remains unknown and controversial, we tested whether BMP-2 could influence PC cell growth, migration and invasion. We previously found [14] that the exposure of endothelial HUVE cells to 5 μM 4HPR caused the release of BMP-2 in the culture medium (50-100 ng/ml concentrations). When DU145 and PC3 cells were exposed to the same BMP-2 concentrations in long-term experiments (4 days and in the absence of 4HPR), cell growth, migration and invasion were significantly decreased in both cell lines (Fig. 6A, B). BMP signaling downregulates the β-catenin pathway in cancer cells [30,31]. While negative regulation on the β-catenin pathway by BMP signaling has been recognized to have a role in intestinal tumorigenesis in mice and humans [30,31] information is lacking about the relationships between the two pathways in prostate tumors. Western blot analysis of nuclear extracts from PC3 cells exposed for 4 days to BMP-2 (50-100 ng/ml) showed a dose-dependent decrease of nuclear β-catenin (Fig. 6C). We then looked at the possible mechanisms controlling β-catenin accumulation. We found that BMP-2 modulates AKT phosphorylation (Fig. 6C), but not that of pGSK3β (data not shown), further confirming that in prostate cancer cells β-catenin nuclear signaling is mainly controlled by AKT activity. As β-catenin promotes transcription of the proliferation gene cyclin D1, we also noted that BMP-2-treated cells exhibited significantly lower levels of cyclin D1 (Fig. 6D).

E-cadherin enforces cell-cell contacts forming the adherens junctions and is anchored to actin filaments by β and α catenin. E-cadherin loss promotes metastasis by enabling the first step of the metastatic cascade: the disaggregation of cancer cells from each another. BMP-7, another member of the BMP family, has been reported to be a potent inhibitor of prostate cancer metastasis [32] also acting through E-cadherin induction. PC3 cells exposed to BMP-2 exhibited increased levels of E-cadherin (Fig. 6D) suggesting that BMP-7 and BMP-2 have similar mechanisms of action. We cannot exclude, however, that β-catenin downregulation controls E-cadherin expression as reported for other compounds [33].

## Discussion

Prostate cancer is the most frequently diagnosed cancer in men and a leading cause of cancer death. Although the 5-year survival rate is excellent for localized stages, the survival dramatically decreases when prostate cancer metastasizes. Decades of research have revealed that cancer is easier to prevent than to treat and for individuals at a high risk of developing cancer and/or for slow evolving cancers, such as prostate cancer, chemoprevention is a logical approach. Preneoplastic lesions such as high-grade prostatic intraepithelial neoplasia (PIN) are frequently observed in asymptomatic young men and it is believed that such lesions require two to three decades to develop into clinically relevant prostate cancer. The fact that prostate cancer is associated with advanced age again suggests that chemopreventive agents inhibiting or delaying the onset of malignancy might be recommended. Identification of molecular targets and signaling pathways of chemoprevention are therefore relevant to cancer therapy.

Several studies have reported a remarkable preventive activity of 4HPR in animal models of prostate cancer [2-6], but pilot clinical trials gave less encouraging results. One possible explanation for the contrasting results may be related to the low dose used in humans compared to those that were effective in animal studies or to the low bioavailability of 4HPR at the prostate tissue level as documented in biopsies [34]. The elucidation of molecular pathways activated by 4HPR are fundamental clues to understand how this agent might be better used in a prevention setting and current trials are underway to re-examine both dose and schedule of 4HPR administration as well as the target tissues of interest.

Our study was designed to investigate the early effects of 4HPR on activated pathways, and regulated gene products, that control prostate cancer cell migration, invasion and proliferation later on. We found that 4HPR inhibited phosphorylated FAK, a protein tyrosine kinase localized at the cell membrane that signals through the PI3K/AKT pathway. Increasing levels of FAK in human prostate cancer correlate with greater metastatic potential [35]. FAK overexpression appeared in PIN lesions, with a clear distribution of high staining in neoplastic cells, while normal cells in the surrounding tissue did not show elevated expression [36]. Furthermore, benign prostate hyperplasia did not show a change in FAK expression compared to normal tissue [36]. These observations support a role for FAK in the pre-metastasis phenotype. We provide evidence that the FAK-mediated decrease of AKT activity by 4HPR treatment, as well as AKT inactivation/activation with Wortmannin or LY294002 and IGF-I or Myr.Akt, respectively, tightly controls the chemotactic and metastatic phenotype of androgen-independent prostate carcinoma cells and may also explain the already described suppression of constitutive NF-*k*B activation, mediating invasion and osteoclastogenesis, in human prostate cancer cells exposed to 4HPR [12,37]. Moreover, as both FAK and AKT signaling controls prostate tumor angiogenesis by up-regulating vascular endothelial growth factor [22,38], our results showing reduction of VEGF release by 4HPR could be associated with FAK and AKT decreased activity.

The Wnt signaling pathway and its key component β-catenin play critical roles in embryonic development as well as in human diseases, including various malignancies. Accumulated evidence has demonstrated a significant role for the Wnt pathway in the development and progression of human prostate cancer. In the absence of a Wnt signal, β-catenin is constitutively down-regulated by a multicomponent phosphorylation destruction complex containing active GSK3β and targeted for degradation by the ubiquitin proteasome pathway. Stimulation of the Wnt pathway results in increased levels of nuclear β-catenin, which activates target genes (i.e cyclinD1) promoting G_1_-S transition and cell cycling. High levels of Wnt and β-catenin are associated with advanced, metastatic, hormone-refractory prostate carcinoma [24]. Blockade of β-catenin signaling by chemopreventive agents suppresses prostate carcinogenesis and metastasis in TRAMP mice and decreased proliferation and invasiveness in DU145 cells, similar to that obtained with siRNA directed against β-catenin [33]. Our data show that both DU145 and PC3 cell lines have high basal levels of soluble β-catenin indicative of an active Wnt signaling. We cannot exclude that 4HPR treatment, besides decreasing AKT phosphorylation, leads to β-catenin degradation by affecting other pathways inducing GSK3β phosphorylation such as Wnt- and ERK-mediated signaling.

Retinoids and the synthetic derivative 4HPR regulate gene expression through the RAR/RXR nuclear receptor family (NR). Retinoid-activated RAR and RXR are potent repressors of β-catenin signaling in retinoid-sensitive cells [39-41] and retinoid-mediated repression of several Wnt genes has been implicated as a required step in the differentiation of neuronal cells [42]. These mechanisms may further contribute to the effectiveness of 4HPR as a chemopreventive agent in cancers with hyperactive Wnt signaling. Moreover, as β-catenin has the ability to enhance androgen receptor (AR) function in prostate cancer [43], the obvious therapeutic goal to abrogate potential oncogenic AR/β-catenin interactions can be easily achieved through the chemopreventive properties of 4HPR also in hormone responsive cells.

Prostate cancer has the ability to produce angiogenic factors and several studies showed that an increased microvessel density is associated with poorer prognosis. We and others [4,12-16,44,45] have previously demonstrated that one possible mechanism of the chemopreventive activity of 4HPR is through inhibition of angiogenesis and invasion, in part mediated by BMP-2 production [14]. In long-term experiments, prostate cancer cells exposed to BMP-2 concentrations attainable in vitro (50-100 ng/ml) from endothelial cells exposed to 5 μM 4HPR, showed a slight but significant decreased proliferation and reduced chemotactic and invasive activities. These effects again associate with decreased AKT activity and lower levels of β-catenin and cyclin D1, indicative of an interference with the β-catenin pathway, as already described in intestinal tumorigenesis in mice and humans [30,31]. Of note, BMP-2 treatment also induced E-cadherin expression, indicative of a less metastatic phenotype. The role of BMPs in the formation of prostate cancer metastasis to bone remains unknown as demonstrated by the great number of published contrasting results. BMP-2, 4, 6 and 7 have in fact been shown to both induce and prevent bone metastasis [32,46-51]. These contrasting results may be generated by the different experimental approaches utilized, time of exposure and concentrations of BMPs employed. We indeed obtained enhanced migration and invasion only when the cells were exposed to BMP-2 during the 5 hours of the assay (data not shown).

## Conclusion

A large number of evidences point into the same direction: FAK, and its downstream signaling molecules AKT and GSK-3β, β-catenin and its upstream and downstream signaling molecules Wnt and cyclin D1, respectively, are important players in both prostate tumor development and metastasis. Simultaneous manipulation by the chemopreventive 4HPR of a number of signaling pathways, both in cancer and endothelial cells, all involved in the processes of tumor progression and metastasis formation is likely to be more effective than manipulation of single target molecules. Investigation of Wnt signaling molecules and identification of synergisms between 4HPR and other candidate chemopreventive molecules with complementary mechanisms of action may support future assessment of this prototype cancer preventive retinoid as an anti metastatic drug.

## Competing interests

The authors declare that they have no competing interests.

## Authors' contributions

RB designed parts of the study, generated the stable cell lines and helped to draft the manuscript, SM and RV carried out protein studies, chemotaxis assays and performed knock-down experiments, FT provided valuable reagents and contributed to the critical revision of the manuscript and the statistical analysis, NF designed the experiments, supervised the project and wrote the manuscript. All authors read and approved the final manuscript.

## Supplementary Material

Additional file 1 **ROS generation by 4HPR. NAC inhibits 4HPR-induced ROS production**. DU145 and PC3 cells treated with 5 βM alone for 1 h, or pretreated for 30 min with NAC at 10 mM were stained with dichlorofluorescein diacetate and analyzed by spectrofluorimetry to assess intracellular ROS production. The significant 4HPR-induced ROS production relative to controls (***P < 0.001) is inhibited by pretreatment with NAC (**P < 0.01). NAC alone produced a marginal effect on the basal ROS level. Click here for file

Additional file 2 **4HPR resistant DU145/R5 cells show an altered signaling**. Inhibition of cell growth (panel A) and migration (panel B) by 4HPR is abolished in DU145/R5 resistant cells. As compared to parental cells, DU145/R5 cells show high level of phosphorylated AKT unrelated to 4HPR exposition (panel C). Click here for file
