# Supplementary figures and images for: The chemopreventive retinoid 4HPR impairs prostate cancer cell migration and invasion by interfering with FAK/AKT/GSK3β pathway and β-catenin stability
Source: Mol Cancer. 2010 Jun 10;9:142. doi: 10.1186/1476-4598-9-142 (PMC2898704; doi:10.1186/1476-4598-9-142)

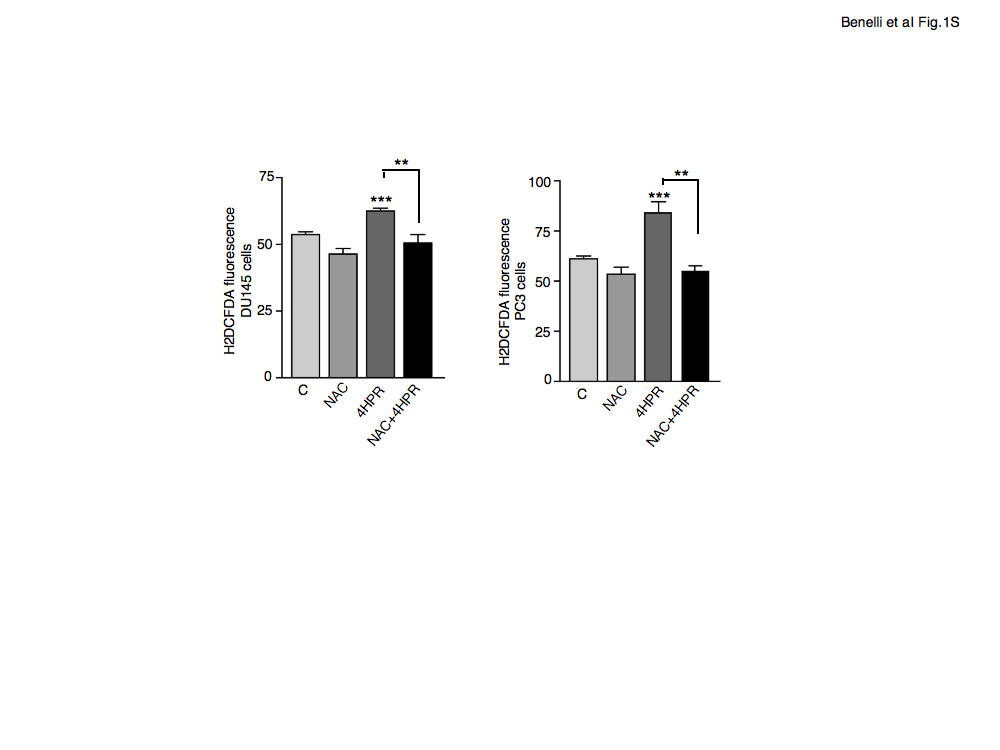

Supplement: Additional file 1 — ROS generation by 4HPR. NAC inhibits 4HPR-induced ROS production. DU145 and PC3 cells treated with 5 βM alone for 1 h, or pretreated for 30 min with NAC at 10 mM were stained with dichlorofluorescein diacetate and analyzed by spectrofluorimetry to assess intracellular ROS production. The significant 4HPR-induced ROS production relative to controls (***P < 0.001) is inhibited by pretreatment with NAC (**P < 0.01). NAC alone produced a marginal effect on the basal ROS level. [file 1476-4598-9-142-S1.TIFF]

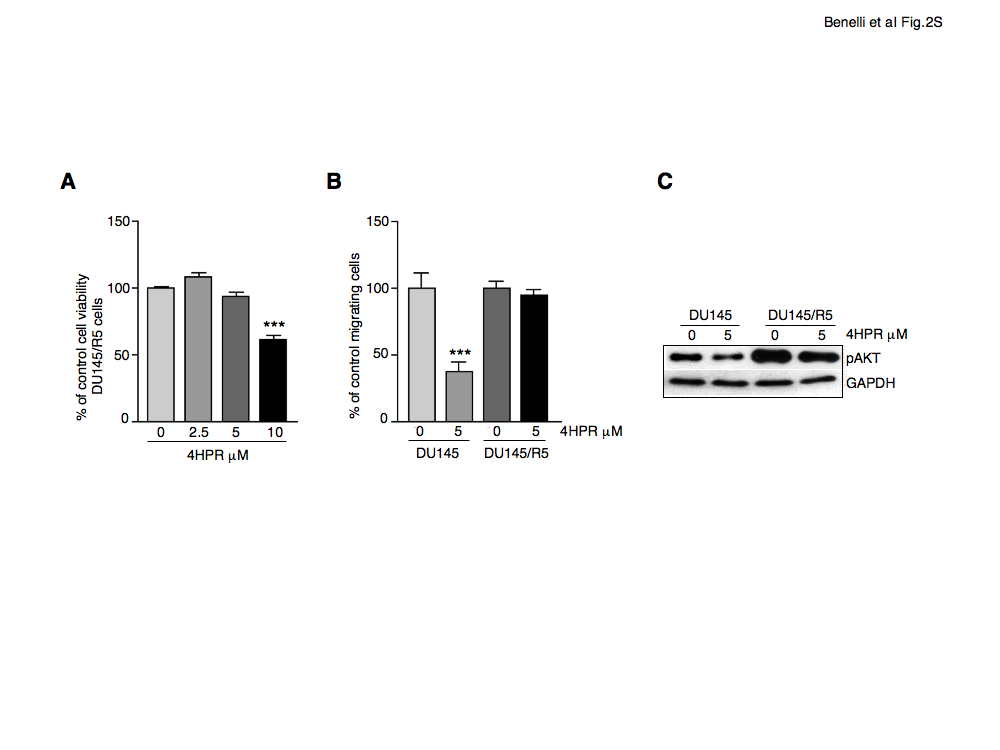

Supplement: Additional file 2 — 4HPR resistant DU145/R5 cells show an altered signaling. Inhibition of cell growth (panel A) and migration (panel B) by 4HPR is abolished in DU145/R5 resistant cells. As compared to parental cells, DU145/R5 cells show high level of phosphorylated AKT unrelated to 4HPR exposition (panel C). [file 1476-4598-9-142-S2.TIFF]
